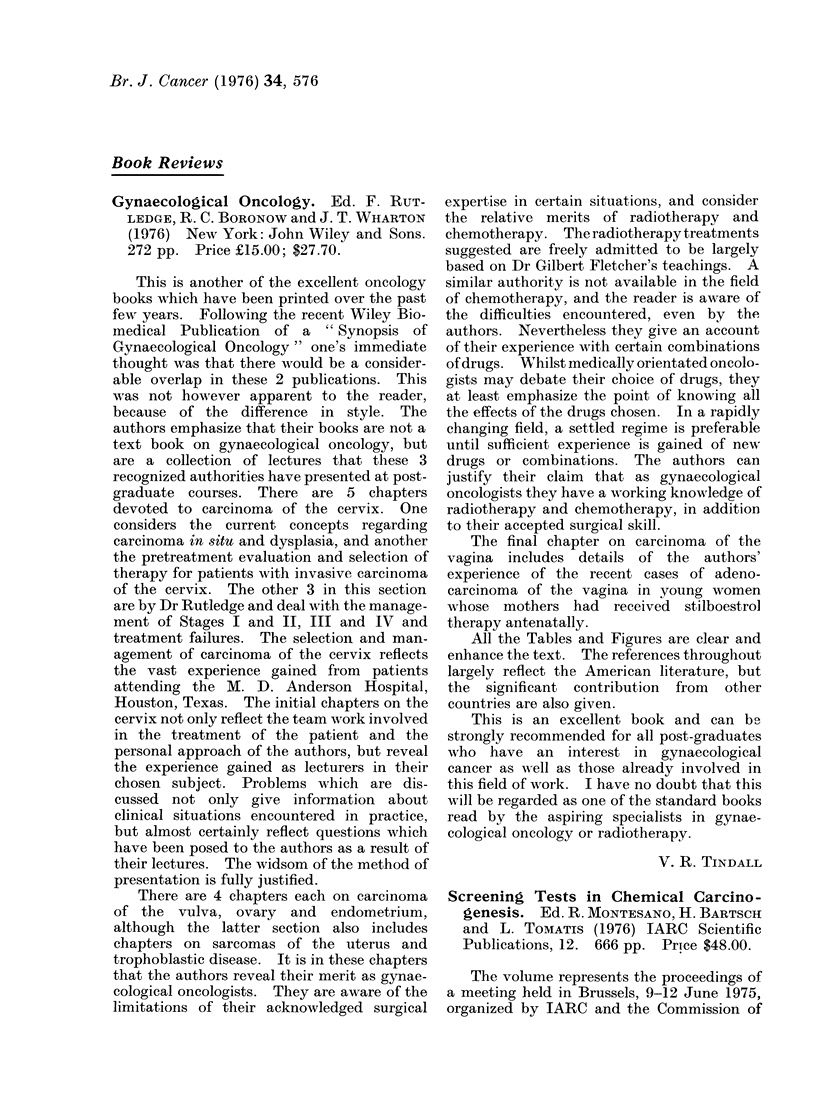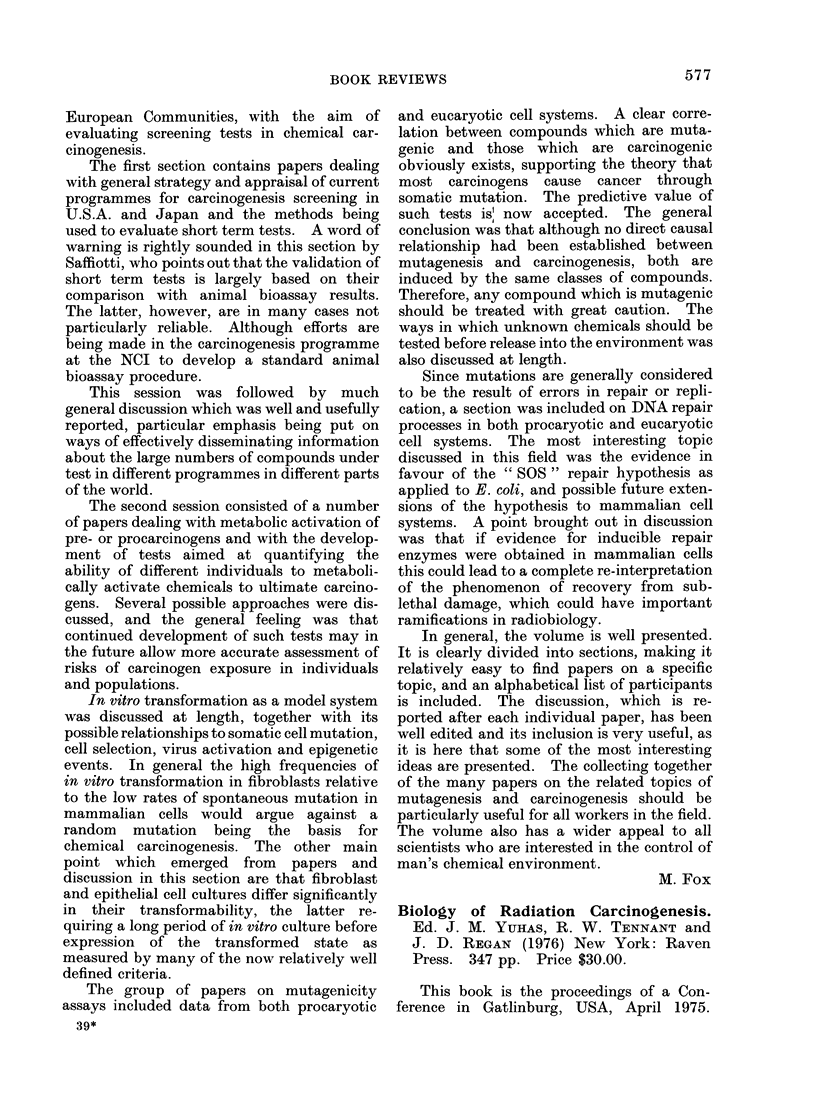# Screening Tests in Chemical Carcinogenesis

**Published:** 1976-11

**Authors:** M. Fox


**Screening Tests in Chemical Carcinogenesis.** Ed. R. Montesano, H. Bartsch and L. Tomatis (1976) IARC Scientific Publications, 12. 666 pp. Price $48.00.

The volume represents the proceedings of a meeting held in Brussels, 9–12 June 1975, organized by IARC and the Commission of

European Communities, with the aim of evaluating screening tests in chemical carcinogenesis.

The first section contains papers dealing with general strategy and appraisal of current programmes for carcinogenesis screening in U.S.A. and Japan and the methods being used to evaluate short term tests. A word of warning is rightly sounded in this section by Saffiotti, who points out that the validation of short term tests is largely based on their comparison with animal bioassay results. The latter, however, are in many cases not particularly reliable. Although efforts are being made in the carcinogenesis programme at the NCI to develop a standard animal bioassay procedure.

This session was followed by much general discussion which was well and usefully reported, particular emphasis being put on ways of effectively disseminating information about the large numbers of compounds under test in different programmes in different parts of the world.

The second session consisted of a number of papers dealing with metabolic activation of pre- or procarcinogens and with the development of tests aimed at quantifying the ability of different individuals to metabolically activate chemicals to ultimate carcinogens. Several possible approaches were discussed, and the general feeling was that continued development of such tests may in the future allow more accurate assessment of risks of carcinogen exposure in individuals and populations.

*In vitro* transformation as a model system was discussed at length, together with its possible relationships to somatic cell mutation, cell selection, virus activation and epigenetic events. In general the high frequencies of *in vitro* transformation in fibroblasts relative to the low rates of spontaneous mutation in mammalian cells would argue against a random mutation being the basis for chemical carcinogenesis. The other main point which emerged from papers and discussion in this section are that fibroblast and epithelial cell cultures differ significantly in their transformability, the latter requiring a long period of *in vitro* culture before expression of the transformed state as measured by many of the now relatively well defined criteria.

The group of papers on mutagenicity assays included data from both procaryotic and eucaryotic cell systems. A clear correlation between compounds which are mutagenic and those which are carcinogenic obviously exists, supporting the theory that most carcinogens cause cancer through somatic mutation. The predictive value of such tests is now accepted. The general conclusion was that although no direct causal relationship had been established between mutagenesis and carcinogenesis, both are induced by the same classes of compounds. Therefore, any compound which is mutagenic should be treated with great caution. The ways in which unknown chemicals should be tested before release into the environment was also discussed at length.

Since mutations are generally considered to be the result of errors in repair or replication, a section was included on DNA repair processes in both procaryotic and eucaryotic cell systems. The most interesting topic discussed in this field was the evidence in favour of the " SOS " repair hypothesis as applied to *E. coli*, and possible future extensions of the hypothesis to mammalian cell systems. A point brought out in discussion was that if evidence for inducible repair enzymes were obtained in mammalian cells this could lead to a complete re-interpretation of the phenomenon of recovery from sublethal damage, which could have important ramifications in radiobiology.

In general, the volume is well presented. It is clearly divided into sections, making it relatively easy to find papers on a specific topic, and an alphabetical list of participants is included. The discussion, which is reported after each individual paper, has been well edited and its inclusion is very useful, as it is here that some of the most interesting ideas are presented. The collecting together of the many papers on the related topics of mutagenesis and carcinogenesis should be particularly useful for all workers in the field. The volume also has a wider appeal to all scientists who are interested in the control of man's chemical environment.

M. Fox